# Metabolomic and Transcriptomic Analyses Reveal Changes in Active Components During the Growth and Development of Comfrey (*Symphytum officinale* L.)

**DOI:** 10.3390/plants14142088

**Published:** 2025-07-08

**Authors:** Jia Fu, Yuqian Liu, Wenting Gou, Mengxue Liu, Nanyi Zhang, Qiang Si, Hongmei Shang

**Affiliations:** 1 College of Forestry and Grassland Science, Jilin Agricultural University, Changchun 130118, China; 15948312048@163.com (J.F.); 18222677958@163.com (Y.L.); 19996256788@163.com (W.G.); 18304317546@163.com (M.L.); zhangny282@nenu.edu.cn (N.Z.); 2 Jilin Provincial Key Laboratory of Tree and Grass Genetics and Breeding, Jilin Agricultural University, Changchun 130118, China

**Keywords:** comfrey, growth periods, flavonoids, phenolic acids

## Abstract

Comfrey (*Symphytum officinale* L.) is a traditional medicinal plant, and its growth period has an important effect on the accumulation of active components. Phenolic acids and flavonoids are the most important active components in comfrey, but their accumulation in comfrey has not been studied. At present, most research on comfrey focuses on its roots. There is still a lack of systematic research on the comparison of active components and biological activities in the aerial parts of comfrey in different growth periods. To explore the influence of the growth period on the active components of comfrey, non-targeted metabolomics and transcriptomics were used to comprehensively analyze the active components of comfrey during the vegetative period, blooming period, and maturity period and compare the dynamic changes in phenolic acid and flavonoid accumulation during different growth periods of comfrey. The results revealed that the vegetative period presented the highest total phenol and flavonoid contents. The predominant secondary metabolites associated with phenolic acids and flavonoids were integral to the phenylpropanoid, flavonoid, and isoflavonoid biosynthetic pathways. Critical structural genes governing these metabolic processes—PAL, C4H, 4CL, CHS, FLS, and DFR—exhibited marked upregulation during the vegetative growth stage. Comprehensive transcriptomic analysis and weighted gene co-expression network analysis were used to construct a co-expression network of structural genes and transcription factors that affected the accumulation of specific metabolites, and the transcription factors related to the synthesis of flavonoids and phenols were predicted. These findings elucidate the temporal regulatory mechanisms governing the growth-phase-dependent accumulation of bioactive constituents in comfrey, advancing the understanding of phytochemical dynamics in medicinal plants.

## 1. Introduction

Comfrey (*Symphytum officinale* L.) is a perennial herbaceous plant in the Boraginaceae family of *Symphytum* [1]. It is widely used to treat diarrhea, bronchitis, tuberculosis, and ulcers [2]. Comfrey extracts can promote wound healing, reduce inflammation, and relieve joint and muscle pain caused by trauma [3,4,5]. Phenolic compounds, alkaloids, and flavonoids such as caffeic acid oligomers (globoidnan A, globoidnan B, rabdosiin, and rosmarinic acid), rosmarinic acid, and salvianolic acid have been recognized as the key active ingredients in the pharmacological effects of comfrey [6]. Relevant studies have shown the ability of comfrey extracts to exhibit biological activities, such as antioxidant [7], antibacterial [8], and anti-inflammatory [9] activities. There are reports on comfrey root extracts’ antioxidant potential [10], immunomodulatory capacity [11], and modulation of human skin microorganisms [2], but relatively little attention has been given to the aerial parts of comfrey.

In recent years, significant interest has focused on understanding the dynamic changes in the accumulation of active substances in various medicinal plants during growth periods. According to diverse studies, the period of growth that coincides with the moment of plant harvest affects the chemical composition, such as the total phenols and flavonoids, as well as the biological activities of the plant [12]. Analysis of *Lycium barbarum* L. berries revealed maximal flavonoid concentrations (0.19 ± 0.006 mg quercetin equivalent/g) and antioxidant activity (0.03 ± 0.001 mmol Fe^2+^ equivalent/g) during the initial phase of fruit ripening [13]. Among the six different growth stages of three-year-old *Lonicera japonica* plants, the contents of phenolic acids and flavonoids were highest in silvery flowers, whole white alabastrum, and white alabastrum [14]. The phenolic compound levels in global artichoke (*Cynara cardunculus* L. var. *scolymus* L.) have been shown to increase with the month of harvest and peak in April [15]. The phenolic acid and flavonoid contents in the aerial parts of comfrey and the dynamic changes occurring during growth and developmental periods remain unelucidated.

With the development of multiomics, the combination of transcriptome, metabolome, and modern molecular biology techniques has become increasingly popular in plant research. Through the integration of metabolomics and transcriptomics, gene expression and metabolite levels can be analyzed in depth during the dynamic development of plant bodies, thereby revealing the biosynthetic pathways of key metabolites and identifying the regulatory factors at play. By integrating the transcriptome and metabolome, Wu et al. [16] identified relevant metabolites and genes in immature and mature blackberry fruits and clarified the molecular mechanism of flavonoid biosynthesis. Through metabolomics, transcriptomics, and proteomics, Ma et al. [17] conducted a comprehensive analysis of the differences in carbohydrate composition and secondary metabolites between mature and postharvest goji berries (*Lycium barbarum* L.), elucidating the metabolic transformation pathways involved and identifying the key regulatory factors leading to these changes. Wang et al. [18] employed integrated transcriptomic and metabolomic analyses to elucidate the differential metabolites and differentially expressed genes (DEGs) associated with the glucosinolate and soluble sugar biosynthesis pathways in Chinese cabbage and identified important transcription factors (TFs) that regulate these two biosynthesis pathways.

This study hypothesizes that the growth period will alter the expression of phenolic acid and flavonoid metabolites and genes, resulting in different transcriptional regulation of related synthetic pathways, thereby affecting the accumulation of active substances and the exertion of biological activities. Therefore, attention should be paid to the three main growth and development periods of comfrey (vegetation (V), blooming (B), and maturity (M)). In order to determine the changes in phenolic acid and flavonoid components in the aerial components of comfrey, the metabolites and transcriptome profiles of comfrey in different growth and development periods were analyzed, thereby predicting the key regulatory networks of phenolic acid and flavonoid biosynthesis. Through a comprehensive analysis of non-targeted metabolomics and transcriptomics, as well as a weighted gene co-expression network analysis (WGCNA), the temporal variations in bioactive constituents throughout the ontogenetic progression of comfrey were elucidated for the first time, thereby establishing a robust framework for the evidence-based assessment and strategic exploitation of comfrey’s superior phytopharmaceutical properties.

## 2. Results

### 2.1. Effects of Different Growth Periods on the Contents of Active Components in Comfrey

Flavonoids and phenolic acids, recognized as critical bioactive constituents in medicinal plants, demonstrate well-documented anti-inflammatory [19] and antioxidant [20] properties. These phytochemicals are integral to mediating physiological processes such as plant growth regulation, developmental modulation, and adaptive responses to environmental stressors [21] and are key components of comfrey for anti-inflammatory effects, pain relief, and wound healing [22].

**Figure 1:**
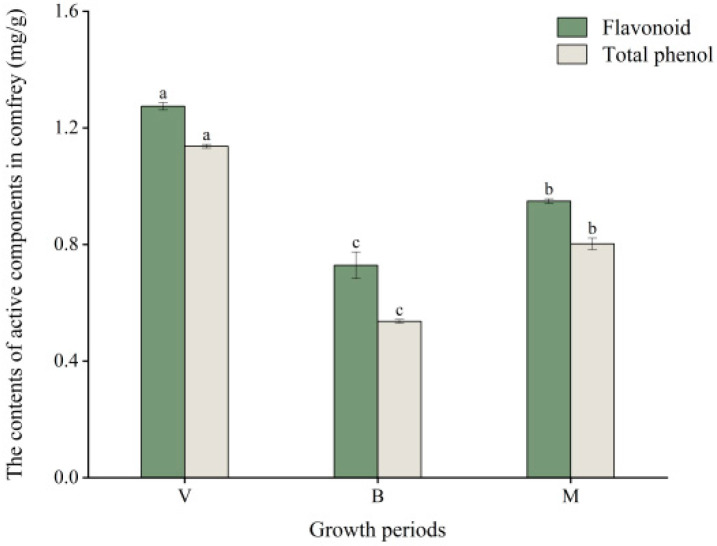
The contents of active components and antioxidant activities of comfrey at different growth periods. V, vegetative period; B, blooming period; and M, maturity period. Different letters (a–c) marked on the bar chart of the same index indicating significant difference (*p* < 0.05).

The concentrations of phenolic acids and flavonoids in comfrey were quantified across developmental periods, revealing dynamic variations in both secondary metabolites (Figure 1). The maximum concentrations of flavonoids and phenolic acids were observed in V (1.27 mg/g, 1.14 mg/g), demonstrating statistically significant differences compared to M (0.95 mg/g, 0.80 mg/g) and B (0.73 mg/g, 0.54 mg/g) (*p* < 0.05). Therefore, V may be the key period for the accumulation of active components in comfrey, and comfrey can be harvested in V. These results confirm that the dynamic changes in the active components of comfrey are closely related to the development period, so metabolome and transcriptome analyses were performed during different development periods of comfrey.

### 2.2. Metabolome and Differentially Accumulated Metabolites (DAMs) Analyses

**Figure 2:**
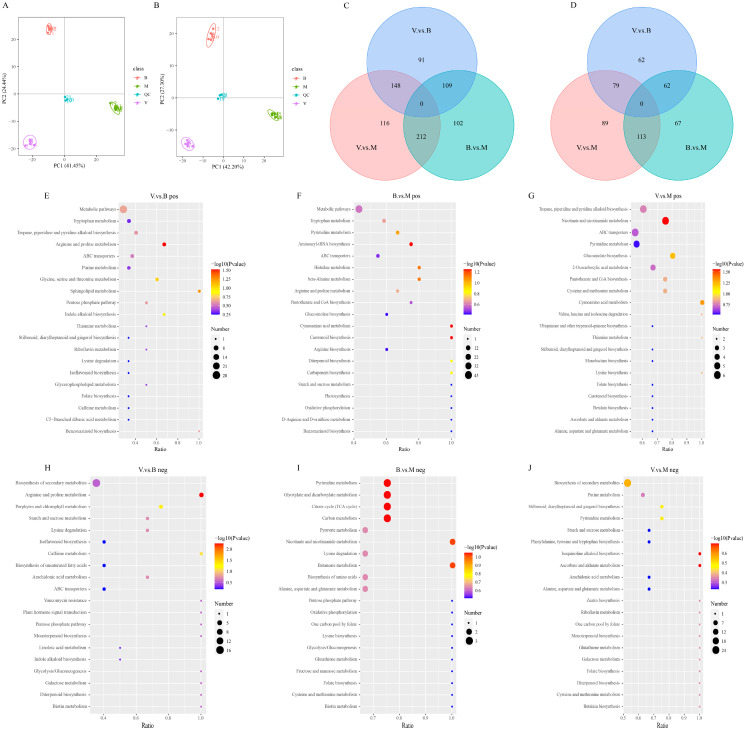
Metabolome analysis for comfrey at different growth periods. (**A**,**B**) Principal component analysis of total samples under the mode of positive and negative ions. (**C**,**D**) Venn diagram analysis of differential metabolites under the mode of positive and negative ions. (**E**–**J**) Kyoto Encyclopedia of Genes and Genomes enrichment analysis under the mode of positive and negative ions. V, vegetative period; B, blooming period; M, maturity period; QC, quality control.

In total, 1120 and 675 metabolites were identified in the three periods of comfrey growth, among which the first six categories were lipids and lipid-like molecules, phenylpropanoids and polyketides, organic acids and derivatives, organoheterocyclic compounds, benzenoids, and organic oxygen compounds under positive and negative ion modes (Figure S1). The results of the principal component analysis (PCA) of the total metabolite samples and quality control samples during the growth process of comfrey are shown in Figure 2A,B. The three samples clearly separated under the positive and negative ion modes, which indicates that the development period had a significant influence on the DAMs. The DAMs were appraised based on the projection value variable importance derived from partial least squares discriminant analysis (PLS-DA). In the positive ion mode, 778 DAMs were identified in total, and 348 (190 upregulated and 158 downregulated), 476 (324 upregulated and 152 downregulated), and 423 (287 upregulated and 136 downregulated) DAMs were identified in V vs. B, B vs. M, and V vs. M, respectively; under negative ion mode, a total of 472 DAMs were identified, and 203 (86 upregulated and 117 downregulated), 281 (161 upregulated and 120 downregulated), and 242 (150 upregulated and 92 downregulated) species of DAMs were found in the three comparison groups, as shown in Figure 2C,D. All DAM information between the comparison groups under the two modes is provided in Table S1.

Metabolic pathways related to DAMs in each comparison group were screened by Kyoto Encyclopedia of Genes and Genomes (KEGG) enrichment analysis. The most enriched KEGG pathways were the biosynthesis of other secondary metabolites, amino acid metabolism, the metabolism of cofactors and vitamins, nucleotide metabolism, and carbohydrate metabolism. Most of these enriched pathways were related to secondary metabolite synthesis and amino acid metabolism (Figure S2). Significantly enriched pathways were screened according to *p* < 0.05 (Figure 2E–J). The DAMs in the V vs. B periods were significantly enriched in arginine and proline metabolism under the positive and negative ion modes. Cyanoamino acid metabolism and nicotinate and nicotinamide metabolism were the pathways associated with the marked enrichment of DAMs in the V vs. M periods in the positive ion mode. There was no significant enrichment pathway for B vs. M. These results indicate that the DAMs in these coenriched pathways may be closely related to the biological activities of comfrey and can be used as the main way to study differences in chemical composition during different growth periods.

### 2.3. Transcriptome and DEG Analyses

**Figure 3:**
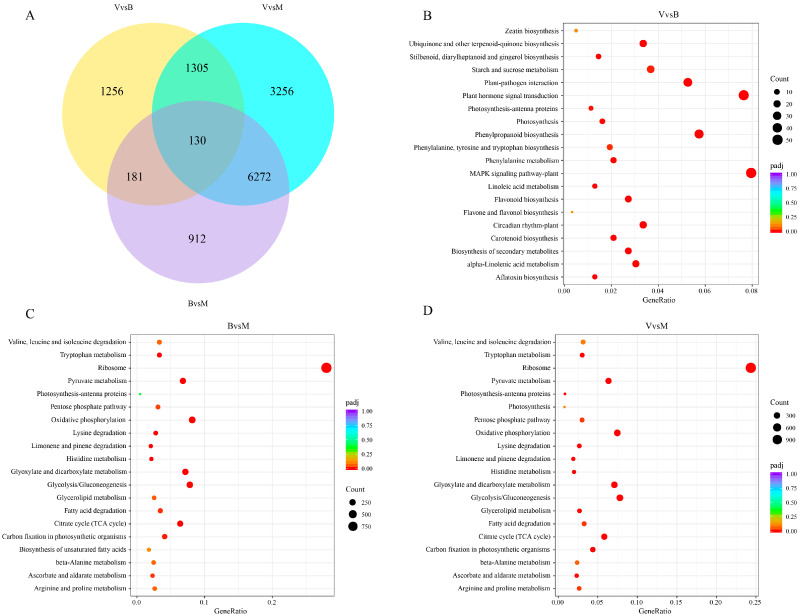
For comfrey in different growth periods. (**A**) Venn diagram analysis of differential expressed genes. (**B**–**D**) Kyoto Encyclopedia of Genes and Genomes enrichment analysis. V, vegetative period; B, blooming period; and M, maturity period.

To explore the molecular reactions linked to the detected metabolic alterations, RNA-seq analysis was carried out on comfrey leaves in various developmental periods. From the nine samples in the constructed complementary DNA (cDNA) library, 197 million clean reads were obtained. The percentage of Q30 (the proportion of nucleotides with mass values above 30) in the transcriptome data spanned from 95.47% to 98.04%, and the GC content ranged from 42.79% to 46.10% (Table S2). These findings suggest that the transcriptome data are trustworthy and can be utilized for further annotation and differential expression analysis. At present, there is no nucleotide record of comfrey in the NCBI nucleotide database, and no reference genome is available. Therefore, Trinity technology was used to splice the RNA-seq data, and RSEM software was used to compare the obtained transcriptome as a reference sequence with the clean reads of each sample. Reads with comparison mass values less than 10 were filtered out. According to the DESeq2 identification results, the DEGs associated with comfrey in different developmental periods were screened, and 13312 DEGs were identified by consensus (Table S3). In V vs. B, B vs. M, and V vs. M, there were 2872 (2117 upregulated and 755 downregulated), 10,963 (2514 upregulated and 8449 downregulated) and 7495 (756 upregulated and 6739 downregulated) DEGs identified, respectively (Figure S3); among these DEGs, 1305, 181, and 6272 DEGs were found in any two of the three comparison groups, and 130 DEGs were found in all three comparison groups (Figure 3A). These findings demonstrate distinct transcriptional profiles in comfrey during distinct developmental phases, with the observed DEGs exhibiting dynamic expression patterns correlated with ontogenetic progression.

Furthermore, KEGG pathway enrichment analysis was conducted to delineate biologically significant pathways associated with the identified DEGs within each experimental group. When *p* < 0.05, significantly enriched pathways were confirmed for each group (Figure 3B–D, Table S4). These pathways fell into the following four broad categories: metabolism, environmental information processing, organismal systems, and genetic information processing. The main category of “metabolism” contained the most DEGs, especially “carbohydrate metabolism” (2661 DEGs), “energy metabolism” (1019 DEGs), and “amino acid metabolism” (771 DEGs). Among the five pathways with the highest degree of significant enrichment in the V vs. B group, three pathways were related to the biosynthesis of other secondary metabolites, namely, phenylpropanoid biosynthesis, flavonoid biosynthesis, and the biosynthesis of secondary metabolites. The top five pathways significantly enriched in the B vs. M and V vs. M groups were carbohydrate metabolism, translation, and the metabolism of terpenoids and polyketides, and a total of four pathways were related to carbohydrate metabolism, namely, glycolysis/gluconeogenesis, the citrate cycle (TCA cycle), pyruvate metabolism, and glyoxylate and dicarboxylate metabolism. The identified biological pathways are potentially implicated in the biosynthesis of DAMs across the three distinct experimental periods. The DEGs involved may have potential roles in regulating secondary metabolites.

### 2.4. Integrated Metabolomic and Transcriptomic Profiling

To better explore the molecular mechanism of the accumulation of active components during different growth periods, a combined analysis of metabolism and the transcriptome was performed. The enriched biological pathways at both the metabolome and transcriptome levels among the groups in the two ion modes are shown in Table S5. In the positive ion mode, seven pathways, ABC transporters, aminoacyl-tRNA biosynthesis, beta-alanine metabolism, galactose metabolism, glycine, serine and threonine metabolism, thiamine metabolism, and belladonna, tropane, piperidine, and pyridine alkaloid biosynthesis were enriched in the main DAMs and DEGs of the three comparison groups. ABC transporters and linoleic acid metabolism were the main DAM and DEG enrichment pathways in the three comparison groups under the negative ion mode. These biological pathways may be the main reasons for the differences in the contents of active ingredients in comfrey during the three growth periods. Based on the KEGG pathway enrichment analysis and physicochemical indices data, the biosynthetic pathways associated with flavonoid and phenolic acid synthesis were prioritized for further investigation.

#### 2.4.1. Phenolic Acid Biosynthetic Pathway

**Figure 4:**
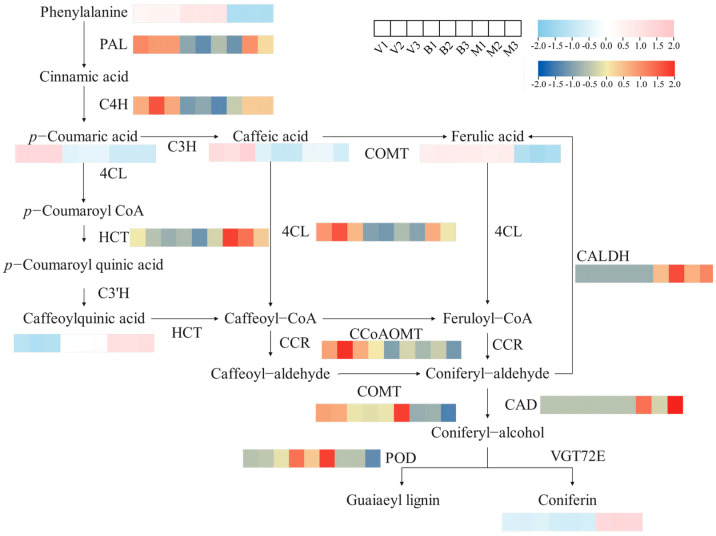
Synthetic analysis of phenols at different growth periods of comfrey. The heat map was generated by TBtools (version 0.665) from the FPKM standardized log2 conversion count, where red indicates up-regulation and blue indicates down-regulation. V, vegetative period; B, blooming period; and M, maturity period.

Phenylpropane biosynthesis constitutes a central metabolic route responsible for the generation of phenolic-acid-derived secondary metabolites within plant systems [23]. Among the three comparison groups, 56 DEGs and 8 DAMs were associated with phenylpropane biosynthesis (Table S6), including key genes regulating phenolic acid biosynthesis. Heatmap combinations of the structural genes and metabolites involved in phenolic acid synthesis were generated based on the KEGG results (Figure 4). With the growth and development of comfrey, the content of common phenolic acids such as caffeic acid, *p*-coumaric acid, and ferulic acid decreased and the content of caffeoylquinic acid increased. In the three periods, the expression of several structural genes involved in phenolic acid biosynthesis, including phenylalanine ammonia-lyase (PAL), cinnamate 4-hydroxylase (C4H), 4-coumarate-CoA ligase (4CL), and caffeoyl-CoA O-methyltransferase (CCoAOMT), was downregulated, and the highest expression level was detected in V. Phenylalanine is converted to *p*-coumaric acid under the action of PAL and C4H. The expression patterns of PAL and C4H in comfrey were similar to the accumulation trend of *p*-coumaric acid. Ferulic acid showed a stable accumulation pattern in V and B and decreased in M, which was consistent with the expression of caffeic acid 3-O-methyltransferase (COMT). The expression of hydroxycinnamoyl transferase (HCT) matched the accumulation pattern of caffeoylquinic acid and gradually increased. The expression patterns of structural genes aligned with their respective metabolite levels, indicating that these genes play a central regulatory role in biosynthetic pathways. The accumulation of phenolic acids in the V samples may be attributed mainly to the relatively high expression levels of PAL, C4H, and 4CL in V.

#### 2.4.2. Flavonoid Biosynthetic Pathway

**Figure 5:**
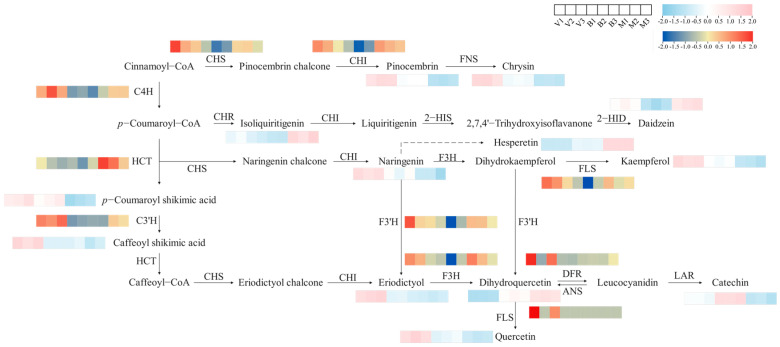
Synthetic analysis of flavonoids at different growth periods of comfrey. The heatmap was generated by TBtools (version 0.665) from the FPKM standardized log2 conversion count, where red indicates upregulation and blue indicates downregulation. V, vegetative period; B, blooming period; and M, maturity period.

Flavonoids regulate plant growth and development. The metabolic pathways related to flavonoid synthesis included flavonoid biosynthesis and isoflavonoid biosynthesis, which included 20 DEGs and 9 DAMs (Table S7). The changes in metabolites and structural genes involved in flavonoid synthesis were systematically mapped via KEGG analysis (Figure 5). Upstream of the pathway, C4H, chalcone synthase (CHS), and chalcone isomerase (CHI) all showed high expression levels in V. Different chalcones are catalyzed by CHI to produce pinocembrin, naringenin, and eriodictyol, three important flavonoids that were also highly expressed in V. Although no related genes encoding flavone synthase (FNS) were found among the DEGs, the accumulation pattern of chrysin paralleled that of pinocembrin, suggesting that pinocembrin might serve as a key precursor for chrysin. Naringenin is catalyzed by naringenin 3-dioxygenase (F3H) to produce dihydrokaempferol, an important hub in the flavonoid biosynthesis pathway, followed by kaempferol under the action of flavonol synthase (FLS). F3H can also catalyze the formation of dihydroquercetin. However, dihydroquercetin accumulation tended to be downregulated in V and upregulated in M, which was inconsistent with the expression pattern of F3H, indicating that other genes may be involved and that further study is needed. Dihydroquercetin is reduced by dihydroflavonol 4-reductase (DFR) to produce leucocyanidin, which is subsequently metabolized by leucoanthocyanidin reductase (LAR) to produce catechins. Dihydroquercetin can also be synthesized under the action of FLS. In addition, several DAMs involved in isoflavonoid biosynthesis, including isoliquiritigenin, daidzein, and hesperetin, presented relatively high levels in M. These findings suggest that pivotal genes in this metabolic sequence (chalcone reductase (CHR), chalcone isomerase (CHI), 2-hydroxyisoflavanone synthase (2-HIS), and 2-hydroxyisoflavanone dehydratase (HIDH)) exert critical regulatory roles in isoflavone biosynthesis, with the isoflavonoid pathway likely serving as the primary biosynthetic route governing flavonoid accumulation in M. Comparative analyses revealed that the transcriptional activity of structural genes and concomitant metabolite concentrations within the flavonoid synthesis pathway were significantly elevated in V relative to other periods. The high concentration of flavonol (kaempferol and quercetin) substances and the apparent upregulation of four DEGs (C4H, CHS, FLS, and DFR) in V may be reasons for the relatively high flavonoid content in V. These findings indicate that the investigated metabolites and structural genes appear to play critical roles in modulating the biosynthesis and accumulation of distinct flavonoid compounds.

### 2.5. Identification of Potential TFs Involved in Core Metabolic Pathways and WGCNA

**Figure 6:**
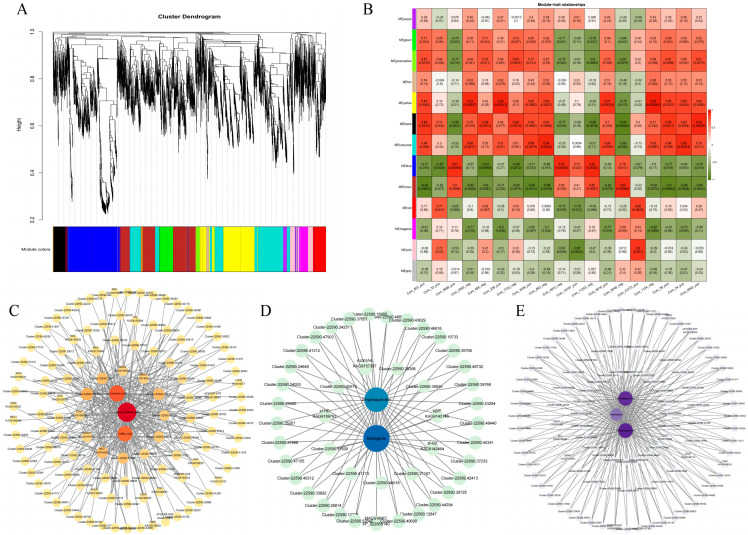
Co-expression network analysis. (**A**) Hierarchical cluster tree. (**B**) Matrix of module–metabolite associations. Each column represents a specific phenols and flavonoids compound (details in Table S2). Correlation coefficients and *p*-values between modules and metabolites are shown at the row–column intersections. (**C**–**E**) Co-expression subnetwork analysis of the yellow, black, brown modules.

WGCNA was performed on all DEGs to further explore the potential association between gene expression and metabolite accumulation dynamics. A total of 13 modules were identified according to the expression profile (Figure 6A). Each color represents a module, and grey represents genes that do not fit into any module. The genes in each module are shown in Table S8, and the associations between these network modules and 19 related flavonoids and phenolic acid metabolites were analyzed. A total of six modules, including yellow, black, turquoise, blue, brown, and pink modules, were strongly associated (Pearson value > 0.9) with related metabolites (Figure 6B). The yellow module showed a significant positive correlation with *p*-coumaric acid (com_2593_neg), caffeic acid (com_236_pos), eriodictyol (com_8518_pos), and caffeoylshikimic acid (com_1154_neg). The black module was positively correlated with naringenin (com_6642_pos) and negatively correlated with dihydroquercetin (com_8549_neg). The turquoise module was positively correlated with chrysin (com_883_neg) and quercetin (com_24_pos). The blue module was positively correlated with coniferin (com_5689_pos), isoliquiritigenin (com_4912_neg), and hesperetin (com_11253_neg) and negatively correlated with ferulic acid (com_488_neg). The brown module was positively correlated with dihydroquercetin (com_8549_neg) and negatively correlated with caffeoylquinic acid (com_603_pos) and kaempferol (com_58_pos). The pink module was highly positively correlated with catechin (com_21273_pos).

Considering the characteristic gene link (KME) value and GS value, the yellow, black, and brown modules were selected as the next analysis modules. In accordance with KME > 0.95, GS > 0.95, weight threshold > 0.45, KME > 0.9, GS > 0.9, and weight threshold > 0.4, black and brown module genes were screened to construct regulatory networks, and potential TFs were identified (Figure 6C–E, Table S9). There were twelve TFs in the yellow module (AP2/ERF-ERF, BES1, bHLH, C2H2, C3H, GRAS, HMG, HSF, MYB, NAC, Tify, and WRKY). The top seven most active gene families were AP2/ERF-ERF, C2H2, bHLH, C3H, HSF, NAC, and Tify. KAG9138893 and KAG9140224, encoding AP2/ERF-ERF TFs, and KAG9160935 and KAG9160935, encoding C2H2 TFs, were found to be associated with three central metabolites, *p*-coumaric acid, and caffeic acid, and caffeoylshikimic acid, with a strong positive correlation (Pearson value > 0.9). A total of four TFs (AUX/IAA, bZIP, MADS-MIKC, and zf-HD) were found in the black module, among which the zf-HD TF was the most active, and KAG9142464 and KAG9159793, encoding the zf-HD TF, were strongly positively correlated with the central metabolite naringenin. In the brown module, seven TFs (AP2/ERF-ERF, bHLH, bZIP, GRAS, MADS-M-type, SET, and WRKY) were found. KAG9146770 and KAG9149155, encoding the TRAF TF, were strongly positively correlated with dihydroquercetin. AP2/ERF-ERF, C2H2, zf-HD, and TRAF may positively regulate the expression of flavonoids and phenolic acid metabolites, potentially mediating accumulation in comfrey.

### 2.6. Quantitative Real-Time PCR (qRT-PCR) Validation

**Figure 7:**
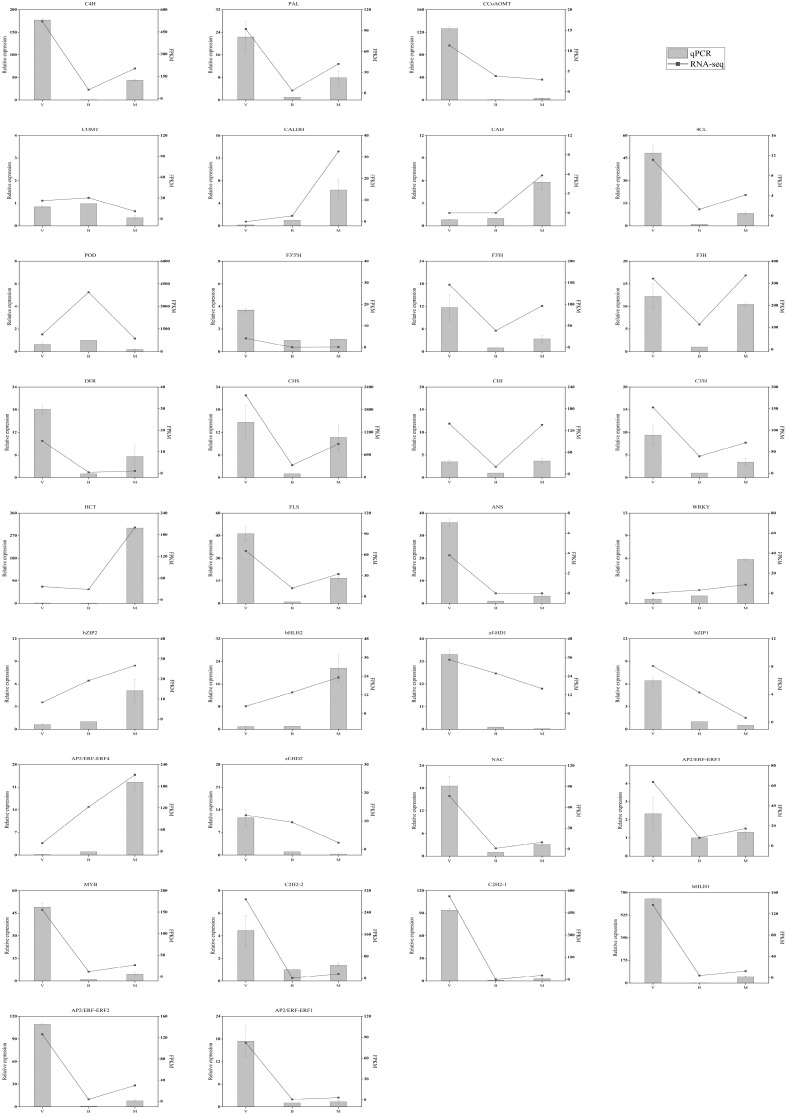
Quantitative real-time PCR (qRT-PCR) validation of 33 structural genes and transcription factors involved in core metabolic pathways. The bar chart and line chart, respectively, reflect the expression profile of qRT-PCR and the trend of RNA-seq. Each experiment featured three biological replicates, with each replicate being duplicated three times for precision. V, vegetative period; B, blooming period; and M, maturity period.

To corroborate the reliability of the transcriptomic data, qRT-PCR was performed on 33 pivotal structural genes and TFs implicated in the phenolic acid and flavonoid biosynthesis pathways. All the expression profiles obtained by qRT-PCR exhibited a high degree of consistency with the transcriptomic trends, thereby corroborating the precision of the RNA sequencing analyses (Figure 7). The consistency between the two analytical approaches strengthens confidence in the dataset’s reliability.

## 3. Discussion

Research on bioactive substances in medicinal plants has increasingly become the focus of global attention. Comfrey, a traditional medicinal plant, has broad application prospects. Comfrey is rich in biological activity, especially high antioxidant activity and anti-inflammatory activity [9,24]. At present, caffeic acid oligomers (globoidnan A, globoidnan B, rabdosiin, and rosmarinic acid) are the main components of comfrey root extracts that exert anti-inflammatory effects [11]. Trifan et al. [25] reported that phenolic compounds such as rosmarinic acid and salvianolic acid were the main contributors to the antioxidant activity of comfrey roots. These findings suggest that the biological activity of comfrey roots is attributable mainly to phenolic derivatives (phenolic acids and flavonoids). However, studies on extracts from the aerial parts of comfrey have been neglected, and the key active determinants and molecular mechanisms of comfrey have not been fully clarified. The accumulation of phenolic acids and flavonoids in the aerial parts of comfrey has not been reported. Furthermore, the biosynthesis of plant secondary metabolites is a highly dynamic and regulated process, and its accumulation pattern profoundly reflects the physiological state of plants and their strategies for adapting to the environment. The individual development stages of plants are accompanied by significant physiological changes, including alterations in the priority of resource allocation (such as from vegetative growth to reproductive input), fluctuations in defense needs, and differences in responses to environmental signals. These transitions inevitably drive the spatiotemporal reprogramming of secondary metabolic pathways [26]. However, regarding the upper part of the polymerized grassland, especially the patterns of how its key active components (phenolic acids and flavonoids) respond to these intrinsic developmental processes and dynamically accumulate in different physiological stages, have not yet been clarified. Therefore, this study focuses on comfrey at the following three key developmental nodes: V, B, and M. Through an integrated analysis of non-targeted metabolomics and transcriptomics, combined with functional verification, the aim is (1) to analyze the developmental sequential accumulation patterns of phenolic acids and flavonoids; (2) identify the key genes and regulatory factors that drive the specific accumulation of this developmental stage; and (3) from the perspective of plant physiological ecology, explore the potential associations between these accumulation patterns of active components and the physiological needs of plants in different developmental periods (such as growth support, defense enhancement, and reproductive protection).

Phenolic compounds have powerful antioxidant activities and are recognized as the most important active components in comfrey [11]. Our metabolomics analysis revealed that the content of phenolic acids showed significant temporal fluctuations during the development of comfrey. Among them, the aerial parts during the V period exhibited an explosive accumulation of phenolic acids (especially ferulic acid, caffeic acid, and *p*-coumaric acid), significantly higher than that during the B and M periods. This accumulation peak is highly consistent with the vigorous vegetative growth of plants during the V period. Ferulic acid is not only an important antioxidant and defense compound [27], but also a core precursor for lignin biosynthesis. The accumulation of its advantages in the V period is likely to provide the necessary cell-wall-strengthening substances (lignin precursors) for the rapidly growing stem and leaf tissues to support structural formation and mechanical strength. Meanwhile, phenolic acids such as caffeic acid and *p*-coumaric acid, in addition to protecting new tissues from reactive oxygen species damage as antioxidants [28,29], are also widely regarded as participating in the basic defense ‘priming’ of plants [30]. Plant tissues in V are tender and metabolically active, and are vulnerable to invasion by pathogenic bacteria and herbivores. Therefore, the high accumulation of phenolic acids in V can be explained as a comprehensive plant strategy during the rapid growth stage: on the one hand, it meets the metabolic requirements of structure building (such as the flow of ferulic acid to lignin), and on the other hand, it pre-accumulates defense compounds to cope with potential biological stress risks. The analysis of gene expression in the phenylpropane biosynthesis pathway further supports this physiological picture. In V, we observed that the expressions of key enzyme genes in the coding pathways, such as PAL, C4H, 4CL, CCoAOMT, and COMT, were significantly upregulated. The synergistic activation of these genes directly drove the increase in the flux of phenolic acid precursors, providing a molecular basis for the large-scale synthesis of phenolic acids (especially ferulic acid) observed in phase V. It is worth noting that COMT not only participates in the synthesis of lignin monomers, but also in the methylation of certain defensive phenolic acids. The high expression in its V phase implies the synergistic allocation of resources between structural defense (lignin) and chemical defense (specific phenolic acids), jointly serving the protection and support requirements of rapidly growing tissues in V.

Flavonoids are important secondary metabolites produced during plant growth, and extracted flavonoids are also beneficial compounds with antioxidation and anti-tumor effects. In this study, a metabolic pathway map was constructed with the flavonoid and isoflavone biosynthesis pathways as the core, and flavonoid metabolites and genes associated with different growth periods of comfrey were identified. In the study, the changes in flavonoid content during the three growth periods revealed that the growth period significantly affected the content and composition of flavonoids. Most metabolites, including pinocembrin, naringenin, eriodictyol, chrysin, kaempferol, and quercetin, accumulated in V. As a natural flavonoid, pinocembrin has been found to have a variety of pharmacologically active functions and has been shown to be useful in the treatment and prevention of cardiovascular disease and ischemic stroke [31,32]. Naringenin has outstanding antioxidation, anti-diabetes, antidepressant, antiatherosclerosis, and other effects [33,34,35,36]. Eriodictyol and its derivatives are essential for coping with signaling pathways and metabolic disorders in plants [37]. Chrysin is a flavonoid compound that has anticancer and antiestrogenic properties and therapeutic effects on the liver and kidney [38,39]. Kaempferol is widely found in medicinal plants and has powerful health benefits [40]. As an essential flavonol, it has a wide range of antiproliferation, antioxidation, anticancer, and other medicinal properties [41,42]. Quercetin is classified as a flavonoid known for its anti-inflammatory and immunomodulatory properties [43]. These flavonoids with pharmacological activity may be the key components of comfrey for its medicinal value. Growth period effects on phytochemistry, especially flavonoids, have also been observed in other plant species. For example, Vlaisavljevic et al. [44] determined the total flavonoid content of Trifolium pratense L. in three growth stages (30 cm, 50 cm, and bud) and reported that the sample in the vegetative stage (30 cm) presented the highest flavonoid content, including isoflavonoids, daidzein, and genistein. Pretti et al. [45] reported that in aerial parts of Tithonia diversifolia collected from Viana, the total flavonoid content in the vegetative stage was greater than that in the reproductive stage, which may have been due to a shift in the distribution of biosynthesis pathways in the growth and development stages. The alterations in flavonoid metabolites and associated biological processes are intricate. The crucial genes implicated in these biological processes might be regulated either individually or in a synergistic manner by the structural genes located upstream and downstream of the flavonoid synthesis pathway. The expression patterns of genes in the flavonoid biosynthesis pathway are highly consistent with the dynamics of metabolite accumulation. The upstream genes (C4H and CHS) and the key downstream genes (CHI, F3H, flavonoid 3′-hydroxlase (F3’H), DFR, and anthocyanidin synthase (ANS)) generally showed the highest expression levels in V, providing a transcriptional driver for the extensive synthesis of flavonoids in V. It is notable that the relative expression abundances of different branch genes during development may determine the temporal changes in flavonol composition, thereby affecting their physiological functional profiles. For instance, the high expression of FLS in phase V may have directly promoted the accumulation and dominance of flavonols such as kaempferol and quercetin in phase V. Future research needs to further analyze how the fine regulation of these key structural genes precisely matches the specific flavonoid requirements of plants in different developmental stages.

WGCNA is commonly used in plants to identify potential TFs and genes [46,47]. Through WGCNA analysis, we identified that three modules (yellow, black, and brown) were highly correlated with the developmental and temporal accumulation of phenolic acids and flavonoids. These modules were enriched with multiple structural genes involved in the phenylpropane/flavonoid pathway (such as PAL, 4CL, CHS, F3H, and FLS) and 15 core TFs, including members of the AP2/ERF-ERF, bHLH, C2H2, MYB, NAC, bZIP, zf-HD, and WRKY families. The expressions of these TFs showed obvious developmental stage specificity: family members such as AP2/ERF-ERF, bHLH, C2H2, MYB, NAC, and bZIP were generally highly expressed in stage V. This was highly consistent with the explosive accumulation of phenolic acids and flavonoids in phase V, strongly suggesting that they are the core regulatory hubs driving the metabolic program of phenylpropane in phase V. The AP2/ERF-ERF family is involved in stress responses and secondary metabolism [48,49]. Its high expression in phase V may respond to the intrinsic ‘state of tension’ (such as elevated ROS levels) and environmental stress perception related to rapid growth in phase V, thereby activating the synthesis of defensive phenolic substances. The bZIP, MYB, bHLH, WRKY, and NAC families activate structural genes involved in the phenolic acid and flavonoid biosynthesis pathways, such as FLS, PAL, CHI, ANS, 4CL, DFR, and LAR, thereby regulating plant growth and development and adaptation to stress [50,51,52,53,54]. Although there are few reports on the direct regulation of the C2H2 and zf-HD families with phenolic acid/flavonoid accumulation, it is known that they play important roles in organ development (C2H2) and stress response/developmental regulation (zf-HD) [55,56,57,58,59,60,61,62,63,64]. For example, C2H2 members such as AtIDD8 regulate flowering time [59], and zf-HD members are specifically expressed in organs [64]. Their high expression in phase V provides a novel perspective, that is, TFs (such as C2H2 and zf-HD) that regulate organ development and stage transitions may profoundly affect the initiation and intensity of secondary metabolic programs in direct or indirect ways (such as regulating hormone signals and influencing cell proliferation states), thereby closely coupling the accumulation of metabolites with specific developmental stages (such as the rapid vegetative growth period, which requires strong defense). This part explains why phase V becomes the peak of metabolic accumulation—the TF that regulates the developmental stage simultaneously ‘turns on’ the secondary metabolic switch. In contrast, some members of families such as AP2/ERF-ERF, bHLH, bZIP, and WRKY had higher expression levels during M. This might indicate a shift in the focus of metabolic regulation: WRKY usually responds strongly to biological stress, and the increase in its M phase may be related to the demand for enhanced protection of floral organs during the flowering period. Some AP2/ERF or bZIP family members may be involved in hormone signals related to flower development or respond to specific environmental pressures during the flowering period. These TFs may, in turn, regulate the biosynthesis of specific secondary metabolites required in the M phase, such as anthocyanins and specific defense compounds. To sum up, our TF analysis not only revealed the role of known metabolic regulatory factors (AP2/ERF, bHLH, and MYB) in driving the metabolic peak in phase V, but more importantly, discovered a strong association between the TF family (C2H2 and zf-HD) closely related to organ development and stage transition and the accumulation of phenolic acids/flavonoids. This suggests a higher-level regulatory logic: plants precisely coordinate secondary metabolic programs by integrating transcriptional networks that regulate developmental sequences (such as C2H2 and zf-HD) and stress/metabolic responses (such as AP2/ERF, WRKY, and bZIP). They match these with the core physiological needs of different developmental stages. This multi-level transcriptional regulatory network is the core molecular basis for the significant developmental temporal variability of the active components of comfrey.

## 4. Materials and Methods

### 4.1. Reagents and Materials

**Figure 8:**
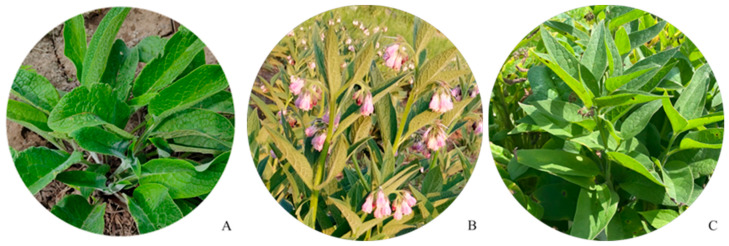
The photos of the aerial parts of comfrey at different growth periods. (**A**), vegetative period; (**B**), blooming period; and (**C**), maturity period.

The following three sampling time points in the pasture garden of Jilin Agricultural University were selected according to the growth period of the aerial parts of comfrey: V (8 June 2023), B (25 June 2023), and M (13 July 2023) (Figure 8 and Table S10). During the growth process of the comfrey, conventional field management such as weeding was carried out without fertilization or the application of environmental factors that would hinder plant growth. Three sample plots of 1 m^2^ each were collected from the aerial parts of comfrey during each period. The comfrey was identified and confirmed by Professor Nanyi Zhang from Jilin Agricultural University. The samples were dried by the hot air-drying method (50 °C) and then pulverized, and the powder obtained after being sieved through a 40-mesh sieve was placed in self-sealing bags and stored at −20 °C in a freezer until subsequent ethanol extraction and analysis. All chemicals utilized were of analytical grade and were employed without further purification.

### 4.2. Preparation of Ethanol Extracts

Ethanol extracts of the aerial parts of comfrey were prepared by an ultrasonic-assisted method [65]. One gram of sample was combined with 25 mL of 95% ethanol, and the mixture was ultrasonicated at 20 °C and 100 W for 60 min by using an ultrasonic processer (KQ-100KDE, Kunshan ultrasonic instruments Co., Ltd., Kunshan, China). The mixture was filtered and immediately stored at −20 °C. The samples were extracted to determine the active ingredient indicators. Samples for metabolomic and transcriptomic analysis were frozen in liquid nitrogen and then transferred for storage at −80 °C. Each experiment was conducted in triplicate.

### 4.3. Determination of Physicochemical Indices

#### 4.3.1. Determination of Flavonoid Content

The quantification of flavonoid content was conducted according to the methodology outlined by Xu et al. [66], with minor adjustments. A rutin standard (0.025 g) was accurately weighed and dissolved in 75% ethanol to prepare a 0.5 mg/mL reference solution, followed by dilution to a final volume of 50 mL. Aliquots (0, 0.4, 0.8, 1.2, 1.6, and 2.0 mL) of a standard solution (0.1 mg/mL) were pipetted into 10 mL graduated test tubes. Each aliquot was diluted with anhydrous ethanol to a volume of 5 mL and vortexed thoroughly. Subsequently, 0.3 mL of 5% NaNO_2_ solution was added, followed by vigorous mixing and incubation for 6 min at ambient temperature. Next, 0.3 mL of 10% Al(NO_3_)_3_ solution was introduced, and the mixtures were agitated and allowed to react for an additional 6 min. Thereafter, 4 mL of 1 mol/L NaOH solution was incorporated, followed by the addition of anhydrous ethanol to adjust the final volume to 10 mL. The solutions were mixed completely for 10 min using a mechanical shaker. A reagent blank, prepared with anhydrous ethanol, served as the reference. Absorbance measurements were conducted at 510 nm using a spectrophotometer, and a calibration curve was constructed by plotting absorbance against concentration. The linear regression equation was *Y* = 0.0182*X* − 0.0154, where *Y* is the absorbance and *X* is the equivalent content of rutin (*r*^2^ = 0.9991). One milliliter of the extract was pipetted into a tube, and the abovementioned reaction procedure was implemented. The absorbance at 510 nm was quantified, and the results are reported as milligrams of rutin equivalent per gram of comfrey. Each experiment was conducted in triplicate.

#### 4.3.2. Determination of Total Phenol Content

The total phenol content was determined by the Folin–Ciocalteu colorimetric method [67]. Gallic acid (GA) was accurately weighed (0.0050 g) and dissolved in distilled water to prepare a standard stock solution (100 μg/mL) in a 50 mL volumetric flask. Aliquots (0, 0.1, 0.2, 0.3, 0.4, and 0.5 mL) of the stock solution were pipetted into separate 10 mL volumetric flasks. Each aliquot was supplemented with 6 mL of distilled water and vortex-mixed for homogenization. Subsequently, 0.5 mL of Folin–Ciocalteu reagent was added, followed by vigorous agitation for 1 min. A 1.5 mL aliquot of 20% Na_2_CO_3_ solution was introduced, and the mixtures were thoroughly blended. The solutions were added to 10 mL with distilled water, then incubated in a thermostatically controlled water bath at 75 °C for 10 min. After cooling to ambient temperature, absorbance measurements were performed at 765 nm using reagent blanks for baseline correction, enabling the construction of the calibration curve. The calibration regression equation was *Y* = 0.0109*X* + 0.0292, where *Y* is the absorbance and *X* is the GA equivalent content (*r*^2^ = 0.9996). An aliquot (0.1 mL) of the extract was subjected to the previously described reaction protocol, and absorbance measurements were recorded. The findings are quantified as milligrams of GA equivalent per gram of comfrey. Each experiment was conducted in triplicate.

### 4.4. Metabolome Analysis

The aerial parts of comfrey (100 mg) were cryogenically pulverized using liquid nitrogen. The resulting homogenate was resuspended in prechilled 80% methanol via vortex agitation, followed by metabolite profiling using ultra-high-performance liquid chromatography–tandem mass spectrometry (UHPLC-MS/MS) at Novogene Co., Ltd. (Beijing, China) [68]. The samples were injected onto a Hypersil Gold column (100 × 2.1 mm, 1.9 μm, Thermo Fisher, Waltham, MA, USA) using a 12-min linear gradient at a flow rate of 0.2 mL/min. The eluents for the positive polarity mode were eluent A (0.1% FA in water) and eluent B (methanol). The eluents for the negative polarity mode were eluent A (5 mM ammonium acetate, pH 9.0) and eluent B (methanol). The solvent gradient was set as follows: 2% B, 1.5 min; 2–85% B, 3 min; 85–100% B, 10 min; 100–2% B, 10.1 min; and 2% B, 12 min. A Q Exactive^TM^ HF mass spectrometer was operated in the positive/negative polarity mode with a spray voltage of 3.5 kV, a capillary temperature of 320 °C, a sheath gas flow rate of 35 psi, an aux gas flow rate of 10 L/min, an S-lens RF level of 60, and an aux gas heater temperature of 350 °C.

The raw data generated by UHPLC-MS/MS were processed using Compound Discoverer 3.3 (CD3.3) to perform peak alignment, peak picking, and quantitation for each metabolite. The mzCloud (https://www.mzcloud.org/, accessed on 6 July 2025), mzVault, and MassList databases were used to obtain accurate qualitative and relative quantitative results. Statistical analyses were performed using the statistical software R (version R-3.4.3), Python (version Python 2.7.6), and CentOS (version CentOS release 6.6). When the data were not normally distributed, they were standardized according to the following formula: sample raw quantitation value/(the sum of sample metabolite quantitation value/the sum of QC1 sample metabolite quantitation values) to obtain relative peak areas. Additionally, compounds with CVs of the relative peak areas in QC samples that were greater than 30% were removed, and finally, the identification and relative quantification results were obtained.

Qualitative and quantitative metabolite analyses, including the annotation and classification of DAMs, were performed in accordance with the protocols established by Yan et al. [69]. The metabolites were annotated using KEGG database, Human Metabolome Database (https://hmdb.ca/metabolites, accessed on 6 July 2025), and LIPID Maps database (http://www.lipidmaps.org/, accessed on 6 July 2025). PCA and PLS-DA were performed with metaX (a flexible and comprehensive software for processing metabolomics data). We applied univariate analysis (*t* test) to calculate the statistical significance (*p* value). Metabolites with a variable importance in projection (VIP) of > 1, *p* < 0.05, and a fold change (FC) of > 1.5 or FC < 0.667 were considered to be DAMs.

### 4.5. RNA Sequencing and Analysis

Total RNA was isolated using the TIANGEN DP441 RNAprep Pure Polysaccharide and Polyphenol Plant Total RNA Extraction Kit (TIANGEN, Beijing, China). RNA integrity was assessed using an Agilent 2100 Bioanalyzer (Agilent Technologies, Palo Alto, CA, USA). Polyadenylated mRNA was enriched via oligo(dT)-conjugated magnetic bead selection and subsequently subjected to fragmentation through random enzymatic cleavage. The first cDNA strand was synthesized with mRNA serving as a template and a random hexamer. The cDNA library was constructed and sequenced using the Illumina HiSeq platform (Illumina, San Diego, CA, USA). Nevertheless, the complete genome assembly and annotation outcomes of comfrey were not attained. Hence, through eukaryotic collateral-free transcriptome sequencing, the raw reads were processed using Fastq. The clean reads were read and spliced by Trinity. The reference transcriptome was assembled de novo using Trinity. High-quality reads from each sample were aligned to the reference transcriptome with RSEM [70]. Differential expression analysis was performed using DESeq2, with significance thresholds set at |log_2_ (FC) |> 1 and adjusted *p*-value < 0.05. Significantly DEGs were functionally annotated through mapping to the Gene Ontology database (http://geneontology.org/, accessed on 6 July 2025) and subjected to pathway enrichment analysis via the KEGG resource (https://www.genome.jp/kegg/pathway.html, accessed on 6 July 2025)

### 4.6. Integrated Metabolomic and Transcriptomic Analysis

Integrated transcriptomic and metabolomic datasets were analyzed by annotating the DEGs and DAMs from corresponding experimental groups to the KEGG database, enabling the identification of co-enriched biological pathways. Subsequent pairwise Pearson correlation analysis between annotated genes and metabolites was performed, with statistically significant associations defined by established thresholds (*R*^2^ > 0.8 and *p* < 0.05).

### 4.7. WGCNA and Gene Network Visualization

The WGCNA TBtools plugin (https://github.com/ShawnWx2019/WGCNA-shinyApp, accessed on 6 July 2025) was used to construct the network, including the network construction module, genetic selection module, and gene function analysis module. All DEGs were initially screened, and genes with undetectable or relatively low expression levels (FPKM < 2) were excluded. The expression values of the remaining 4437 genes were imported into WGCNA, and co-expression modules were constructed using the default automatic network builder function (blockwiseModules). The specific parameters included a power of 23, a minModuleSize of 30, a module cuttree height of 0.25, and the default values of the other parameters were retained (Figure S4). The modular interaction networks were analyzed and graphically represented using Cytoscape (v.3.9.1), a computational software platform designed for network visualization and analysis.

### 4.8. qRT-PCR Analysis

An R6827-01 Plant RNA Kit (Omega, Guangzhou, China) was utilized to extract a total RNA concoction from comfrey leaves during the three different growth periods. Following suit, these RNA extracts were converted into cDNA using the FSQ-101 reverse transcription kit (TOYOBO, Osaka, Japan), as outlined in the manual. Then, we delved into qRT-PCR validation with a Bio-Rad CFX96 system (Bio-Rad Laboratories, Hercules, CA, USA). The PCR was executed with the TBGreen^®^ Premix Ex Taq™ II protocol (TAKARA, Tokyo, Japan). β-Actin was employed as the endogenous control for normalization, and relative mRNA expression levels were quantified via the 2^−ΔΔCt^ method. The primer sequences utilized in the qRT-PCR analysis are comprehensively listed in Table 1. Each experiment featured three biological replicates, with each replicate being duplicated three times for precision.

### 4.9. Statistical Analysis

All experimental data were subjected to statistical evaluation using ANOVA implemented in SPSS 22.0 software (IBM Corp., Endicott, NY, USA). Multiple comparisons of means were performed by Duncan’s multiple range test. Quantitative results are presented as mean ± standard error, with statistical significance defined at *p* < 0.05.

## 5. Conclusions

In this study, the metabolome and transcriptome of the aerial parts of comfrey were comprehensively analyzed and the changes in active components during different growth periods were compared. The results revealed that the phenolic acid and flavonoid contents of V comfrey were the highest. Metabolically, 778 and 472 DAMs were identified in the positive and negative ion modes, respectively. In total, 13,312 DEGs were identified via transcriptomics. Phenylpropane biosynthesis, flavonoid biosynthesis, and isoflavonoid biosynthesis are the main pathways of phenolic acid and flavonoid accumulation. On the basis of joint analysis and WGCNA, the key regulatory networks of phenolic acid and flavonoid biosynthesis were predicted. This study reveals that the accumulation of secondary metabolites (phenolic acids and flavonoids) in comfrey is highly dynamic and stage-specific. High accumulation in period V is a comprehensive strategy for plants to adapt to multiple physiological needs (structural support, antioxidation, and basic defense) during the rapid vegetative growth stage. This temporal pattern is precisely controlled by a complex transcriptional regulatory network, which not only contains the classic metabolic-pathway-specific TFs (MYB and bHLH), but also integrates TFs (C2H2 and zf-HD) that regulate developmental stage transitions and organogenesis and the TFs (AP2/ERF, WRKY, and bZIP) that respond to the environment and stress. This multi-level regulation ensures the synchronization of secondary metabolic programs with the intrinsic developmental clock of plants and changes in the external environment. The research results lay a foundation for preliminary studies on the regulation of bioanabolism by the active components of comfrey and provide a theoretical framework for the sustainable development and management of comfrey resources.

## Figures and Tables

**Table 1:** Specific primers for the qRT-PCR.

*Genes*	Forward Primer Sequence (5′ to 3′)	Reverse Primer Sequence (5′ to 3′)
*β-actin*	TGAGTGGTGGTACGACTATGTTCCC	TCATGCTACTTGGTGCCAATGCTG
*AP2/ERF-ERF1*	AAGAGGCGGCTCGGGCATAC	CGGGCTCGGGATCTCCAGAAG
*AP2/ERF-ERF2*	TGCCTCACGCCTCTGTAGTGTC	GGAACGAAAACCCGCCCTGAAC
*AP2/ERF-ERF3*	TGACGGAGGTTTGGGAAGTTTAGC	AACATTTGACACGGCGGAGGAG
*bHLH1*	TCTGCTTCTGCTTCCACCGTTTC	AATGAGCGTTTGAAGGCGTTGTTG
*C2H2-1*	GTGGTGTTACGGCTTCGGAAGG	GGTTTCTTGGACGGATGAGGACTC
*C2H2-2*	CCTTAGCAGCCGTGGTGGAAATAG	CACCTCATCATCACCGCCAGAAC
*MYB*	CCGCTTGAGAGTAGAGTTCCAGTG	CGGGCTCATGCGAGGTTTGG
*NAC*	TCAGCCAGCCATCCTCTTACTCG	TTGCCATTGAAGGAAGCCCACATC
*bZIP1*	GCTGAAAGGGTGGTCGCTTCTG	GACAGTCTGCTGCTCGATCAAAGG
*zf-HD1*	GGTTCACGGCGGTGGAAGTTG	GACGGCTGCGGTGAGTTCATG
*zf-HD2*	TAGCCTCCGCCATTACCACTACC	AGTTGCAGCCACCGCATTGAG
*AP2/ERF-ERF4*	TGCTGCCCAATCAATCTCCACTG	TGGATTTCTCCGAGGGTGTGTTTG
*bHLH2*	GCCTCATCCACAAGCGACATCTC	TCTACATTCGTTCGGGCATCACAG
*bZIP2*	TGCCATCAGAAACAACTGCCAGAG	GCCTGAGGTGATCGTCTACATTGC
*WRKY*	ATTTGGGGTTACAAGGGCACTCTC	GAGGCATTTGGGCATGTTGTGAAG
*4CL*	ATTTTGCTGGGTAGGGCTGCTTG	AACAGGGTTATGGCATGACGGAAG
*CAD*	CCTTGAAGCCAGCGGAATGTACC	CGAGTCACACTGAGCACCAACAC
*POD*	TTGCAGAAAATGAGGAGGCAGACC	AAAGCAGTGTTCTTGTGGCGTTTG
*CALDH*	GGTCCCCAGGTCTCAAAACTTCAG	ACCCTTGTCACCCTTGCGTTTG
*COMT*	AGAATTTTCCAGCAGGAGCCAACC	AAGAACCCAGAAGCAGCCTCAATG
*CCoAOMT*	GCTCTTTCAATGGCTCTGGCTCTC	ACCCAAGTTTGCTGGTCACAAGG
*PAL*	CTCCCCTGCCTCCCCTTTAGC	AGGGATTGAAGGGAAGCCATTTGG
*C4H*	TCTTCCATTGGGCAGGGTTGTTTG	GCCACACATGAACCTCCACGAC
*CHS*	GTCTCCGCCCTTCTGTCAAACG	CAGCCAAGTCCTTCGCCATACG
*CHI*	CCGTTGTACCTGCCATAGGAGTTG	CCGTCTTTGCCAGGAAGGATTCG
*HCT*	AGACGGGCGGGCTAGGTTTC	AGTTCACCGGCTAATGCGATTGG
*C3’H*	CGGCTTCACTCATTACACGGTCAG	GGACAATGGCAGAACTCCTAAGGC
*F3H*	AGGCAATGGGCTTGGAGAAAGAAG	CTGGCTGAGGGCATTTTGGGTAG
*F3’H*	TCCAGACCGCCTTCCAACCG	TTCGCAGTCGTCTGAGGCAATTC
*F3’5’H*	GGTGCTAAGGCTCTTGGTGACTG	GTGCTCGACTCATACATGGCTTGG
*DFR*	CCCCTTCATCACACCAACATTCCC	AAGGCGGAGTTCTGCAATCTGATG
*ANS*	ACCCAAAATGCCCTCAACCAGAAC	CCAGGAACCATGTTGTGGAGGATG
*FLS*	TGCACACTCACATACCTCTTCC	CGACACCGAGTTAGAATCAAATCC

## Data Availability

The data will be made available on request.

## Supplementary Materials

The following supporting information can be downloaded at: https://www.mdpi.com/article/10.3390/plants14142088/s1, Figure S1: Classification of different metabolite of comfrey at different growth periods under the mode of positive (A) and negative (B) ions; Figure S2: Metabolite pathways and annotation classification of comfrey at different growth periods under the mode of positive (A) and negative (B) ions; Figure S3: The difference compares the number of differentially expressed genes in the combination; Figure S4: Analysis of network topology for various soft-thresholding powers. The upper panel shows the scale-free fit index as the soft-thresholding power; Table S1: Differential metabolites screening at different periods; Table S2: Sample sequencing data quality; Table S3: Differential genes screening at different periods; Table S4: Enrichment of significant pathways in each comparison group at different periods; Table S5: KEGG pathway involving both differential metabolites and differential genes at different periods; Table S6: Differential metabolites and differentially expressed genes associated with phenolic biosynthesis in each comparison group; Table S7: Differential metabolites and differentially expressed genes associated with flavonoid biosynthesis in each comparison group; Table S8: Genes in each module; Table S9: Potential transcription factors in the modules; Table S10: Details of the locations of the three growth periods.
