# Supplementary material for: Metabolomic and Transcriptomic Analyses Reveal Changes in Active Components During the Growth and Development of Comfrey (Symphytum officinale L.)
Source: Plants (Basel). 2025 Jul 8;14(14):2088. doi: 10.3390/plants14142088 (PMC12299076; doi:10.3390/plants14142088)
Supplement: Supplementary file 1 [file plants-14-02088-s001.zip › supplement figure.pdf]

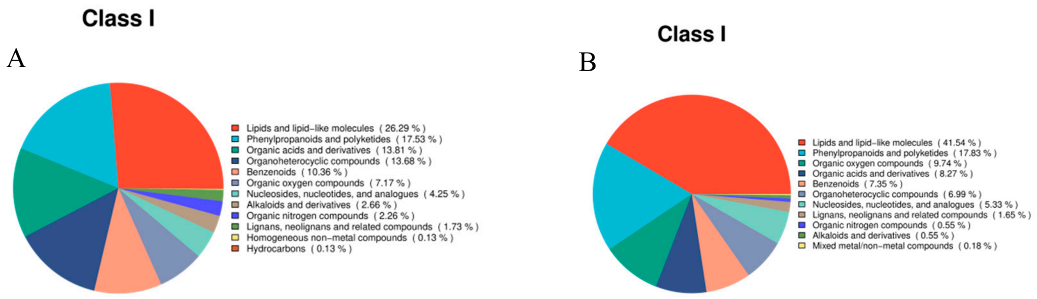

Figure S1. Classification of different metabolite of comfrey at different growth periods under the mode of positive (A) and negative (B) ions.

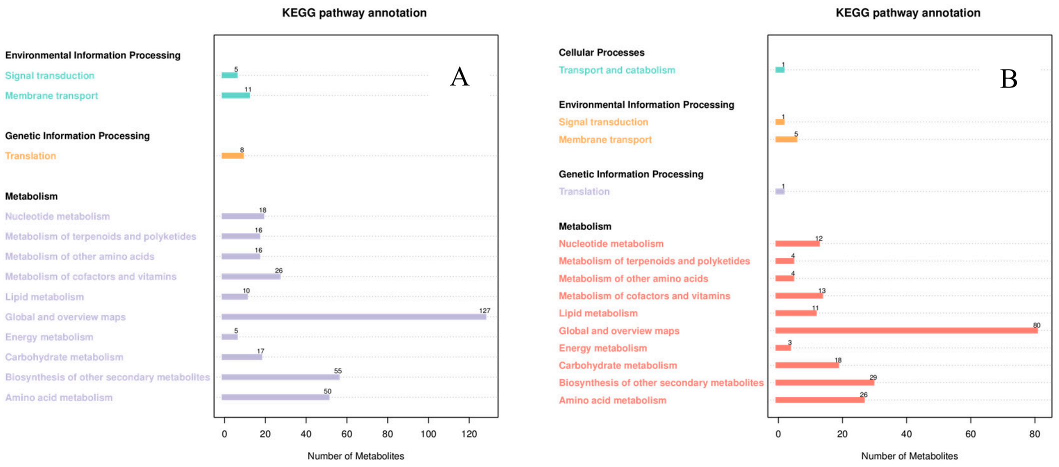

Figure S2. Metabolite pathways and annotation classification of comfrey at different growth periods under the mode of positive (A) and negative (B) ions.

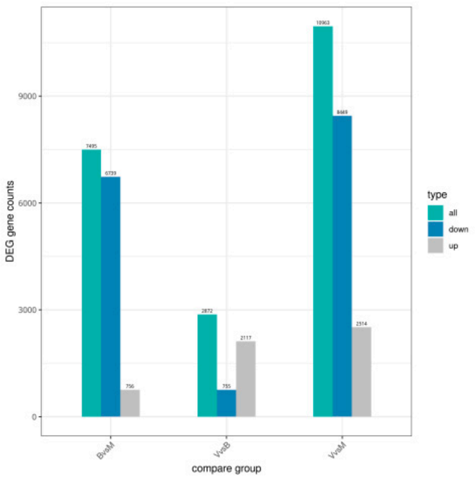

Figure S3. The difference compares the number of differentially expressed genes in the combination. Gray and blue indicate up-regulated and down-regulated differential genes respectively, and the number on the column indicates the number of differential genes; V, vegetative period; B, blooming period; M, maturity period.

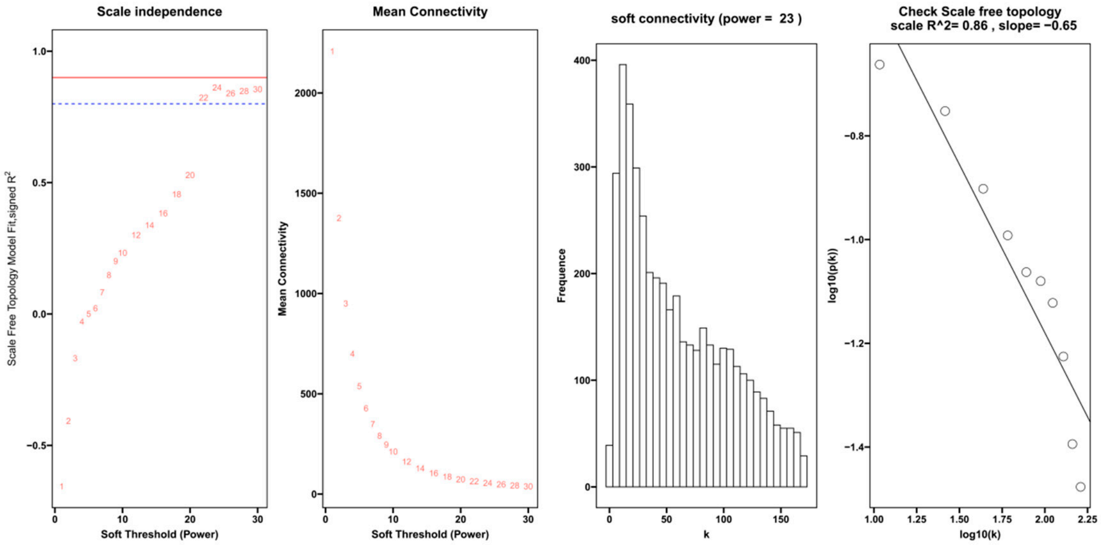

Figure S4. Analysis of network topology for various soft-thresholding powers. The upper panel shows the scale-free fit index as the soft-thresholding power. The mean connectivity (degree, y-axis) as soft-thresholding power was shown in the bottom panel.
